# Individual and mutual effects of diabetes, hypertension, and obesity on acute respiratory distress syndrome mortality rates in clinical patients: a multicentre study

**DOI:** 10.3389/fpubh.2023.1219271

**Published:** 2023-06-21

**Authors:** Márcio Flávio Moura de Araújo, Flávia Paula Magalhães Monteiro, Thiago Moura de Araújo, José Cláudio Garcia Lira Neto, Lívia Fernanda Siqueira Santos, Isaura Letícia Tavares Palmeira Rolim, Floriacy Stabnow Santos, Livia Maia Pascoal, Ana Cristina Pereira de Jesus Costa, Marcelino Santos Neto

**Affiliations:** ^1^Department of Family Health, Oswaldo Cruz Foundation, Eusébio, Brazil; ^2^Health Science Institute, University for International Integration of the Afro Brazilian Lusophony, Redenção, Brazil; ^3^Center for Social Science, Health and Technology, Federal University of Maranhão, Imperatriz, Brazil

**Keywords:** COVID-19, comorbidity, diabetes, obesity, hypertension, epidemiology

## Abstract

Patients with comorbidities are more vulnerable to severe clinical cases of acute respiratory distress syndrome (ARDS) and COVID-19 require complex health care. To analyse the association between the individual and combined effects of diabetes, hypertension, and obesity on ARDS mortality rates among patients receiving clinical care. A multicentre study encompassing retrospective data analysis and conducted with 21,121 patients from 6,723 health services across Brazil, during the 2020–2022 time period. The sample group consisted of clinical patients of both sexes and different age groups who received clinical care and showed at least one comorbidity. The data collected were analysed using binary logistic regressions and the Chi-square test. The overall mortality rate was 38.7%, with a higher predominance among males (*p* < 0.001), mixed-race individuals (*p* < 0.001), and older adults (*p* < 0.001). The main comorbidity variables associated with and leading to death from ARDS were arterial hypertension (*p* < 0.001), diabetes mellitus (*p* < 0.001), diabetes mellitus and arterial hypertension (*p* < 0.001), cardiovascular diseases (*p* < 0.001) and obesity (*p* < 0.001). Both the patients who progressed to recovery (48.4%) and to death (20.5%) presented only one comorbidity (*χ*^2^ (1,749) = 8, *p* < 0.001), respectively. The isolated comorbidities with the greatest impact on death outcomes were diabetes (95% CI 2.48–3.05, *p* < 0.001), followed by obesity (95% CI 1.85–2.41, *p* < 0.001) and hypertension (95% CI 1.05–1.22, *p* < 0.001), even after adjusting for sex and number of simultaneous comorbidities. Diabetes and obesity, as isolated conditions, had a greater influence on the number of deaths of clinical patients with ARDS compared to those with mutual diagnosis of diabetes, hypertension and obesity.

## 1. Introduction

Since the beginning of the pandemic, chronic, non-communicable diseases (NCDs) have been associated with severe and lethal cases of COVID-19, given that NCDs are characterised by a set of pathologies with multiple causes and risk factors, long latency periods, and prolonged courses (1). Coronavirus infections have affected people of different health profiles. However, we have noticed a higher prevalence of severe clinical complications among those affected by pre-existing comorbidities. In this regard, diabetes mellitus, hypertension, and obesity are reported to be among the most lethal NCDs in the presence of viral infections in their most severe form, according to several publications in different scenarios around the world (2–4).

It has been unanimously confirmed by meta-analysis data that cardiovascular diseases, obesity, hypertension, and diabetes are the main comorbidities observed in COVID-19-infected patients (1, 5, 6). In addition, some studies have identified diabetes and hypertension as comorbidities associated with severe and fatal cases, respectively, of COVID-19 and acute respiratory distress syndrome (ARDS) in clinical patients (5, 6). To this end, another review identified that kidney disease is the most prevalent risk factor in cases resulting in patient death, while obesity, even though very prevalent, has shown no association with death in COVID-19 and ARDS cases (1).

As previously mentioned, in isolation, such comorbidities have potentially strong effects in the presence of ARDS, especially in the most severe stage of the disease. However, when these diseases are mutually present in a patient, the combined relationship with the virus, its pathophysiological effects, and other associated factors such as age and sex, is complex and, when consulting the literature, this link seems to not have yet been elucidated.

From this perspective, this study is justified by the need to address and understand the influence between the comorbidities mutually present and patient death outcomes, in order to establish safer and more effective guidelines in the management of hospitalised and/or outpatient ARDS patients. The study of these interactions also has the potential to elucidate mechanisms of hospital readmission for respiratory diseases in humans (7). Therefore, the main aim of this study was to analyse the influence of both the isolated and the combined presence of diabetes mellitus, systemic arterial hypertension, and obesity in ARDS patients on their death rates. We hypothesize that patients with ARDS receiving clinical care affected by these NCDs, either mutually or simultaneously, have a higher death chance in relation to other patients with different comorbidities.

## 2. Materials and methods

### 2.1. Ethical aspects

This investigation was authorized by the Human Research Ethics Committee of the Federal University of Maranhão (UFMA) under approval n° 4,227,396.

### 2.2. Research design

This is a multicentre study using a retrospective data analysis, conducted across 6,723 health services. The service providers are located across 19 healthcare area divisions in the state of Maranhão, including 2,049 primary health care units, 1,246 specialized outpatient clinics, 588 support units (hospitals, diagnosis centres, and treatment centres), and 404 health units. The state of Maranhão is located in the northeast region of Brazil and has approximately 7,075,181 inhabitants living across 217 municipalities. The data used in this study was from a database of patients and were collected between March 2020 and January 2022.

The research analysed the epidemiological surveillance clinical records of the Covid-19 Notification System of Maranhão, Brazil. The system records and tracks notifications of the influenza and the ARDS up to patient death.

The study comprised the period from March 2020 to January 2022, when 386,567 cases of ARDS were registered in the notification system in Maranhão, Brazil. Of these cases, 10,986 resulted in death.

### 2.3. Criteria for defining deaths related to SARS-CoV-2 infection

To define death by ARDS, we considered clinical, epidemiological and/or laboratory criteria.

The clinical criterion consisted of reported deaths of patients diagnosed with acute Covid 19 (clinical picture <7 days) or diagnosed with acute respiratory failure syndrome in a hospital setting. Epidemiological criteria consisted of death of patients who showed close or household contact with a laboratory-confirmed case for COVID-19 in the previous 7 days; death of patients who lived or worked in an area with a high risk of virus spread (nursing homes, homeless shelters, etc.), and who showed up to 7 days of clinical symptoms; or death of a health professional working in a hospital environment with up to 7 days of clinical symptoms. Finally, the laboratory criteria consisted of the death of patients with clinical symptoms in the last 7 days who had undergone the following laboratory tests: molecular biology (detectable positive result for SARS-CoV-2 by the real-time RT-PCR method), or antigen test (reagent for SARS-CoV-2 by the immunochromatography method).

### 2.4. Participants

In this investigation, general patients of both sexes, any age group, with a positive diagnosis for ARDS, and with some comorbidities were eligible. Based on these eligibility criteria, the final sample of this study was 21,121 patients.

### 2.5. Prediction variables

For this study, individual-related variables (sex, age group, race, type of health service, diagnostic criteria, and type of test), as well as pre-existing comorbidities were listed as prediction variables, namely: hypertension, diabetes, diabetes and hypertension, obesity, diabetes and obesity, cardiovascular diseases, respiratory diseases, neurological diseases, cancer, metabolic diseases, kidney diseases, smoking, gastric diseases, psychiatric disorders, HIV-AIDS, rheumatological diseases, autoimmune diseases, hepatitis, alcoholism, urological diseases, diabetes mellitus, leprosy, chemical dependency, haematological diseases, malformation, rare diseases and dermatological diseases.

### 2.6. Outcome variables

The following variables were chosen as outcomes, namely:

- mortality rate in patients with comorbidities,
- mortality rate in patients with diabetes,
- mortality rate in patients with hypertension,
- mortality rate in patients with obesity,
- mortality rate in patients with diabetes and hypertension,
- mortality rate in patients with diabetes and obesity,
- mortality rate in patients with diabetes, hypertension, and obesity.

### 2.7. Data analysis

Uni- and bivariate exploratory analysis of the data were carried out with the aid of the free software JAMOVI version 1.6.

The specific mortality rate from the above selected causes was calculated as follows:


$$\frac{\begin{array}{l} Numberofdeathsofpatientswith\;a\;specificcomorbity\\ betweenJanuary\;2020\;andMarch\;2022 \end{array}}{\begin{array}{l} Totalnumberofdeathsofpatientswith\;any\;comorbity\\ betweenJanuary\;2020\;andMarch\;2022 \end{array}}\times 100$$

The normality of the variables was examined via the Shapiro–Wilk test. We compared the number of commodities per group (deaths vs. recoveries) using the Mann–Whitney test. Through contingency tables, we observed differences in the frequency of comparisons between the predictor variables (comorbidities) and death outcomes (‘yes’ or ‘no’), through a Chi-square test. In turn, for correlations, the Spearman correlation test was used.

In the associations where a *p* < 0.001 were observed, we performed a binary logistic regression analysis, respecting the assumption of the absence of multicollinearity of the variables and the adjustment indices of the models (variance inflation factor [VIF] < 5 and tolerance >0.1). To analyse the goodness of fit for the logistic regression model, we used the Hosmer-Lemeshow test, while to determine the degree of risk, odds ratios were calculated within a 95% confidence interval. Two-tailed Alpha (*α*) values below 0.05 were statistically significant.

## 3. Results

Regarding the profiling of the patients, it can be highlighted that the sample distribution in relation to sex was balanced, but as for the age group, we observed that the older adult group (> 60 years of age) was predominant (54.5%). A substantial portion of the patients had brown skin color, which, in a general Brazilian classification, corresponds to mixed-race individuals. Disease diagnostics and detections took place mostly in public health service providers via laboratory tests, in this case, rapid tests (Table 1).

**Table 1:** Profile of clinical patients with COVID-19 showing at least one comorbidity. Maranhão, Brazil, 2023.

Variable		*n*	%
Age group	0–9	115	0.5
	10–19	295	1.4
	20–29	709	3.4
	30–39	1,766	8.4
	40–49	2,938	14
	50–59	3,813	18
	60–70	4,838	23
	Above 70	6,605	31.5
Sex	Female	10,740	50.8
	Male	10,381	49.2
Race	Brazilian Yellow	2,958	14.9
	Brazilian White	3,409	17.2
	Indigenous	75	0.3
	Mixed race	11,721	59.3
	Black	1,598	8
Death	Yes	8,172	38.7
	No	12,943	61.3
Diagnostic criteria	Clinical examination	119	0.6
	Clinical examination and tomography	192	0.9
	Lab test	20,808	98.5
Laboratory	Public/National	18,821	90.7
	Private	1,915	9.3
Exam type	Serological	404	1.9
	Rapid test	13,813	68.1
	RT-PCR	6,605	30
Health service	Public/National	13,052	88.4
	Private	1,729	11.6

The overall mortality rate was 38.7% in this study. Death cases were higher for men (22.5%) than for women (16.2%) [*χ*^2^(425) =1, *p* < 0.001]. Regarding the group of patient cases who evolved to death, a directly proportional relationship was observed. With an age increase, there is also a statistically significant percentage increase in mortality from ARDS: 0–9 (0.1%), 10–19 (0.1%), 20–29 (0.4%), 30–39 (1.3%), 40–49 (2.6%), 50–59 (4.9%), 60–70 (10%), and > 70 years of age (19.3%) [*χ*^2^ (3024) =8, *p* < 0.001]. Deaths were predominant in mixed raced (24.8%) and white (7.3%) individuals [*χ*^2^ (1357) =5, *p* < 0.001].

Regarding total deaths, the most significant comorbidities for mortality rates were arterial hypertension (63.7%), diabetes mellitus (39.5%), diabetes mellitus and arterial hypertension (26.2%), obesity (7.4%) and diabetes mellitus and obesity (1.8%). The main comorbidity variables associated with and resulting in death from ARDS were arterial hypertension (*p* < 0.001), diabetes mellitus (*p* < 0.001), diabetes mellitus and arterial hypertension (*p* < 0.001), cardiovascular diseases (*p* < 0.001) and obesity (*p* < 0.001) (Table 2).

**Table 2:** Distribution of COVID-19 mortalities among clinical patients with comorbidities. Maranhão, Brazil, 2023.

Comorbidities	Death		
	Yes *n* (%)	No *n* (%)	*p*-value^1^
Hypertension	5,208 (24.6)	7,428 (35.1)	**<0.001**
Diabetes	3,235 (15.3)	2,198 (10.4)	**<0.001**
Diabetes and hypertension	2,142 (10.1)	1,246 (5.9)	**<0.001**
Obesity	612 (2.9)	484 (2.3)	**<0.001**
Diabetes and obesity	149 (0.7)	28 (0.1)	**<0.001**
Diabetes, hypertension and obesity	110 (0.5)	18 (0.1)	**<0.001**
Cardiovascular diseases	1,309 (6.2)	2,565 (12.1)	**<0.001**
Respiratory diseases	543 (2.5)	1,479 (7)	**<0.001**
Neurological diseases	527 (2.5)	192 (0.9)	**<0.001**
Cancer	325 (1.5)	163 (0.7)	**<0.001**
Metabolic diseases	61 (0.2)	86 (0.4)	0.767
Kidney disease	477 (2.2)	312 (1.4)	**<0.001**
Smoking	399 (1.8)	96 (1.4)	**<0.001**
Gastric diseases	14 (0.0)	78 (0.3)	**<0.001**
Psychiatric disorders	52 (0.2)	143 (0.6)	**0.002**
HIV	55 (0.2)	41 (0.1)	**<0.001**
Rheumatological diseases	36 (0.1)	68 (0.3)	0.682
Autoimmune diseases	28 (0.1)	48 (0.2)	0.936
Hepatitis	70 (0.3)	46 (0.2)	**<0.001**
Ethanolism	61 (0.2)	12 (0.0)	**<0.001**
Urological diseases	3 (0.0)	20 (0.0)	0.041
Hansen’s disease	21 (0.0)	19 (0.0)	0.199
Drug addiction	21 (0.0)	407 (1.9)	**<0.001**
Haematological disease	12 (0.0)	24 (0.1)	**<0.001**
Malformation	27 (0.1)	403 (1.9)	**<0.001**
Rare diseases	5 (0.0)	4 (0.0)	0.583
Dermatological diseases	–	10 (0.0)	**0.042**

1 Chi-square test.

The bold values are statistically significant data.

In turn, for the correlation study, we identified that the comorbidities with the highest positive correlation with a death by ARDS outcome were diabetes mellitus (Spearman *rho* = 0.25 e *p* < 0.001), and the simultaneous presence of diabetes mellitus and arterial hypertension (Spearman *rho* = 0.22 e *p* < 0.001). We also observed that death outcomes presented a negative and statistically significant correlation (Spearman rho = −0.27 and *p* < 0.001) with the number of comorbidities per patient. Also, in the 75th percentile, it is possible to verify that the patients who died had 3 simultaneous comorbidities in relation to the group that did not evolve to death, whose comorbidity number was 1 (*p* < 0.001).

**Figure 1:**
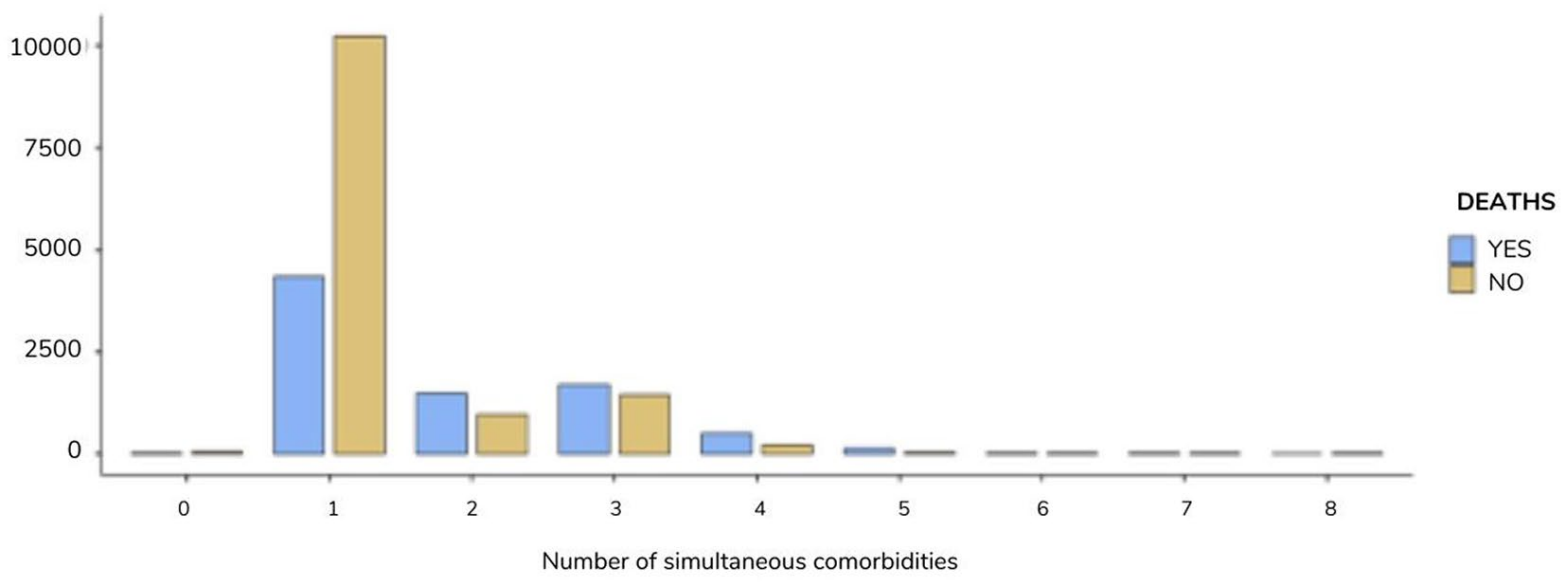
Distribution of the number of simultaneous comorbidities resulting in patient death by ADRS. Maranhão, Brazil, 2023.

We also observed a statistically significant association between death outcome and the number of simultaneous comorbidities. In this case, both those who evolved to recovery (48.4%) and to death (20.5%) had only 1 comorbidity (χ^2^ (1.749) =8, *p* < 0.001), respectively (Figure 1).

When comparing the percentiles for the variable number of comorbidities, we observed that in the 95th and 99th percentiles, the number of comorbidities was 4 and 5, respectively, in the group that evolved to death. In the group that evolved to recovery in the same position, the values found were 3 and 4, respectively (U-Mann Whitney Test, *p* < 0.0001).

The impact of comorbidities on the deaths of ARDS patients was tested in three binary logistic regression models. The most pronounced effect was observed in the comorbidity diabetes (95% CI 2.48–3.05, *p* < 0.001), followed by obesity (95% CI 1.85–2.41, *p* < 0.001) and hypertension (95% CI 1.05–1.22, *p* < 0.001) individually, even after adjustments for sex and number of concurrent comorbidities (Table 3).

**Table 3:** Impact of comorbidities (diabetes, hypertension, and obesity), isolated and/or combined, on the death of clinical patients by ADRS. Maranhão, Brazil, 2023.

Model	Predictor	Odds ratio	Confidence interval		*p*-value
			Lower	Upper	
1	Hypertension	1.30	1.21	1.39	**<0.001**
	Diabetes	3.03	2.73	3.36	**<0.001**
	Obesity	2.60	2.29	2.96	**<0.001**
	Diabetes and hypertension	1.14	1.06	1.30	**0.040**
	Diabetes and obesity	1.38	0.73	2.61	0.319
	Diabetes, hypertension and obesity	1.10	0.51	2.40	0.794
2	Hypertension	1.13	1.05	1.22	**<0.001**
	Diabetes	2.76	2.48	3.06	**<0.001**
	Obesity	2.11	1.85	2.41	**<0.001**
	Diabetes and hypertension	0.51	0.43	0.60	**<0.001**
3	Hypertension	1.13	1.05	1.22	**<0.001**
	Diabetes	2.75	2.48	3.05	**<0.001**
	Obesity	2.11	1.85	2.41	**<0.001**
	Diabetes and hypertension	0.51	0.43	0.60	**<0.001**

Model 1: no fits.

Model 2: fitted for amount of simultaneous comorbidities.

Model 3: fitted for amount of simultaneous comorbidities and sex.

The bold values are statistically significant data.

## 4. Discussion

We found that the isolated presence of diabetes and obesity was more associated with the prevalence of ARDS deaths than the simultaneous presence of comorbidities. By the way, patients with COVID-19 who progress to death used to mostly have at least one comorbidity.

Data from other studies conducted in the Middle East found that the comorbidities most associated with death from COVID-19 were kidney injury, deep vein thrombosis, and tumors (8). Other researchers concluded that the triad of smoking, hypertension, and diabetes mellitus increases the mortality rate (9). In fact, both smoking and kidney injury are mentioned in association with the use of invasive mechanical ventilation, in the case of patients hospitalised for COVID-19 (10).

In this regard, it is important to highlight in this discussion the evidence of a meta-analysis on this topic, which implies that advanced age and the presence of two or more comorbidities are significantly impactful for the clinical evolution of ARDS cases in general hospitalised patients (11). Also, the conclusions of a recent published cohort study are interesting as they show that age and comorbidities can predict the outcome of ARDS, regardless of the severity of the patients’ innate immune response (12). This means that, particularly when NCDs are individually or mutually present in patients, they become an additional burden on the vulnerability of older adults to the SARS-CoV-2. In our findings, we found a positive correlation between death outcomes and the number of comorbidities statistically significant, but a weak association. It was also observed that both in recovered patients and in cases of death, one comorbidity was predominant.

Reviewing the literature, we observed the relationship between the number of comorbidities and mortality from COVID-19 is not unanimously reported, as there are some data that show an exponential relationship, while others show an inverse relationship (13–15). However, the common agreement among the consulted studies is that patients affected by different NCDs and COVID-19 have a high and differentiated prevalence around the world that ranges from 33.8–56.6% of the investigated samples (2).

The inflammatory load related to the COVID-19 pathophysiology is interleukin 6 (IL-6), whose high levels cause cardiovascular damage and the deterioration of the clinical condition of patients, especially of those who already have pre-existing comorbidities, such as chronic NCDs. This is due to these patients having an inadequate immune response owing to other factors such as age, functional impairments, and lifestyle choices (16, 17).

In this sense, fatal cases of ARDS are closely related to the imbalance of the renin-angiotensin-aldosterone system and hyper-inflammation, which causes a dysregulated immune response and a subsequent activation and dysfunction of endothelial cells, which mutually can trigger a thrombotic event. Therefore, in patients with systemic arterial hypertension, for example, this cascade of dysfunctional events can be fatal (18).

Through logistic regression analysis, we detected that mortality rates were higher in those diagnosed with isolated comorbidities of diabetes and obesity, respectively, and the odd ratios for death were more than double and almost doubled (*p* < 0.001) among them. This finding is specifically corroborated by an epidemiological observatory of ARDS related deaths in Brazil, with an interval of 34 weeks of follow-up, which pointed out diabetes and obesity, respectively, always being at the top of the list of factors associated with deaths (19).

In the realm of comorbidities, including hypertension, cardiovascular disease, and chronic obstructive pulmonary disease (COPD), among others, diabetes is considered as a crucial comorbidity for the survival of patients infected by the coronavirus (20). Previous published studies attest to the potent effects of diabetes, in relation to other chronic diseases, to cause deaths by COVID 19 in patients with comorbidities (13, 21). By specifically comparing diabetes and obesity, among the publications consulted, we identified that diabetes is a more potent risk factor for death from COVID 19 (14, 18, 22).

This disease causing a decrease in phagocytic activity, neutrophil chemotaxis, decreased T-cell function, and lower innate immunity in patients affected by Type 2 Diabetes. In addition, among these patients there are higher levels of angiotensin-converting enzyme-2 (ACE2), which serves as an entry receptor for the respiratory virus due to its high binding affinity expressed in pulmonary alveolar cells, cardiac cells, vascular endothelium, as well as other various sites (23).

In the case of obesity, it is well documented in the literature, through observational and review studies, that this condition alone is an intrinsic factor for respiratory failure and that it increases the risk of death in patients affected by COVID-19 by up to five times, as it aggravates previous chronic conditions and increases demand for mechanical ventilation (22–26). Overweight and obesity are characterised by an accumulation of abnormal or excessive fat that increases health risks and can trigger a series of other problems, such as cardiac and vascular conditions, diabetes, endocrinal complications, among others. These are all conditions associated with a high risk of mortality from COVID-19. In addition, the expression of ACE-2 receptors is higher in the visceral adipose tissue than in subcutaneous fat, allowing for a higher viral load and, consequently, more severe forms of the disease in patients with increased visceral fat (4). Inflammatory processes resulting from the infection and immune dysregulation in obese individuals potentialize this relationship (27).

Thus, the scenario presented through our findings poses the pertinent reflection that it is not necessarily the number of comorbidities, but instead, the influence that certain comorbidities can bring to ARDS patients that can lead to an increase in mortality rate.

To date, it is known that the COVID-19 virus binds to the ACE-2, decreasing the activity of this type of receptor and leading to more severe cases. Precisely in patients with diabetes mellitus and systemic arterial hypertension, this receptor is present in high levels. In addition, in the presence of diabetes mellitus, the SARS-CoV-2 virus hijacks an endocrine pathway that plays a crucial role in regulating blood pressure, metabolism, and inflammation, which are aspects that accentuate cellular damage, hyperinflammation, and respiratory failure (28).

In obesity, this bidirectional relationship is no different, given the inflammatory load of the obese and their inefficient immune system in the face of COVID-19 infection. Scholars have stated that cytokine/adipokine levels and inflammatory markers, such as C-reactive proteins, are associated with a higher body mass index in COVID-19-positive patients, suggesting that the inflammatory background and immune dysregulation of obese patients may influence the immune response in this group (27).

Based on our final model, we think it is mandatory for healthcare professionals to consider the amount and type of concurrent comorbidities of patients with ARDS. Therefore, for the purpose of risk stratification, it is crucial for the health care team to understand the parameters that predispose patients with an ARDS to a more severe course of the disease, especially to adopt more appropriate health protocols. In addition, the recognition of these parameters and associated factors are important tools to characterize the typical behavior of the disease, as well as to guide decision-making in the context of public health policies and epidemiological surveillance.

It then becomes clear that the medical team giving care to general patients should adhere to glycemic management and care with nutrition and dietetics focus for people with NCDs and ARDS. This is due to these being triggers for important clinical changes that predict mortality, even when we consider the variable comorbidities. The performance of health workers teams in the management of the above diseases should be mandatory, given that there may be hospital readmission of patients already considered to have recovered from COVID-19 (7). In this sense, the nursing management should include a therapeutic plan focused both on the primary as well as secondary prevention of diabetes and overweight, since many patients may be people with undiagnosed diabetes mellitus or with poor nutritional management.

### 4.1. Limitations

Database-based studies present vulnerability points such as incomplete information or missing data. This was no different in our investigation. For example, we did not have access to the number of days until a death outcome was observed, or to an overview of the current stage of management of the patients’ comorbidities aiming at a possible study of the interaction between the status of said comorbidities and a death outcome. Furthermore, other important data such as vaccination status for COVID-19 and types of medication in use were not part of our database and could provide us with a more complete picture of these patients.

Therefore, we only investigated complete data sheets to avoid the use of *missing data* and thus ensure the best fit of the regression model. In addition, this was a sample of considerable size, which allowed for an in-depth study of the influence of the simultaneous presence of important comorbidities. This investigation may help to generalize and extrapolate findings on mortality rates from ARDS in clinical patients with simultaneous comorbidities, which, to date, lacks in evidence in the current health literature.

## 5. Conclusion

The mortality rates of clinical patients with comorbidities, hospitalised due to ARDS with a simultaneous presence of diabetes, hypertension, and obesity, do not differ from that of those who presented either of these comorbidities individually, or a combination of hypertension and diabetes. On the contrary, a greater impact on death outcomes was observed in patients with the isolated presence of diabetes or obesity.

## Data availability statement

The datasets presented in this study can be found in online repositories. The names of the repository/repositories and accession number(s) can be found in the article/supplementary material. All deaths from COVID-19, obtained from the Maranhão COVID-19 Notification System (SNC-19 MA), linked to the Department of Monitoring and Health Evaluation of the Superintendence of Epidemiology and Disease Control, part of the State Health Department of Maranhão (SES/MA), were considered for the study. This system consolidates data from the notification forms for the flu syndrome and the form for severe acute respiratory syndrome due to COVID-19 (SES/MA, 2020). Data on the population estimate were collected from the IBGE website (https://sidra.ibge.gov.br/). Reference: SES/MA 2020: STATE SECRETARY OF HEALTH OF MARANHÃO (SES/MA). Technical Note No. 01/CIEVS/ /SECD/SAPAPVS/2020. Available at: https://www.saude.ma.gov.br/docs/nota-tecnica-no-01-cievs-secd-sapapvs-2020/.

## Ethics statement

The studies involving human participants were reviewed and approved by Human Research Ethics Committee of the Federal University of Maranhão. The patients/participants provided their written informed consent to participate in this study.

## Author contributions

MA: conceptualization, data curation, formal analysis, funding acquisition, methodology, and writing—original draft. FM and JL: conceptualization, validation, and writing—original draft. TA, LS, IR, FS, LP, AC, and MS: methodology and validation. All authors contributed to the article and approved the submitted version.

## Conflict of interest

The authors declare that the research was conducted in the absence of any commercial or financial relationships that could be construed as a potential conflict of interest.

## Publisher’s note

All claims expressed in this article are solely those of the authors and do not necessarily represent those of their affiliated organizations, or those of the publisher, the editors and the reviewers. Any product that may be evaluated in this article, or claim that may be made by its manufacturer, is not guaranteed or endorsed by the publisher.

## References

[1] Ng WH Tipih T Makoah NA Vermeulen JG Goedhals D Sempa JB. Comorbidities in SARS-CoV-2 patients: a systematic review and Meta-analysis. MBio. (2021) 12:e03647–20. doi: 10.1128/mBio.03647-20, PMID: 33563817 PMC7885108

[2] Richardson S Hirsch JS Narasimhan M Crawford JM McGinn T Davidson KW. Presenting characteristics, comorbidities, and outcomes among 5700 patients hospitalized with COVID-19 in the new York City area [published correction appears in JAMA. 2020 may 26;323(20):2098]. JAMA. (2020) 323:2052–9. doi: 10.1001/jama.2020.6775, PMID: 32320003 PMC7177629

[3] Honardoost M Janani L Aghili R Emami Z Khamseh ME. The association between presence of comorbidities and COVID-19 severity: a systematic review and Meta-analysis. Cerebrovasc Dis. (2021) 50:132–40. doi: 10.1159/000513288, PMID: 33530081 PMC7900456

[4] Vulturar DM Crivii CB Orăsan OH Palade E Buzoianu AD Zehan IG. Obesity impact on SARS-CoV-2 infection: pros and cons "obesity paradox"-a systematic review. J Clin Med. (2022) 11:3844. doi: 10.3390/jcm11133844, PMID: 35807129 PMC9267674

[5] Wang Z Deng H Ou C Liang J Wang Y Jiang M. Clinical symptoms, comorbidities and complications in severe and non-severe patients with COVID-19: a systematic review and meta-analysis without cases duplication. Medicine (Baltimore). (2020) 99:e23327. doi: 10.1097/MD.0000000000023327, PMID: 33235096 PMC7710213

[6] Gold MS Sehayek D Gabrielli S Zhang X McCusker C Ben-Shoshan M. COVID-19 and comorbidities: a systematic review and meta-analysis. Postgrad Med. (2020) 132:749–55. doi: 10.1080/00325481.2020.1786964 32573311

[7] Akbari A Fathabadi A Razmi M Zarifian A Amiri M Ghodsi A. Characteristics, risk factors, and outcomes associated with readmission in COVID-19 patients: a systematic review and meta-analysis. Am J Emerg Med. (2022) 52:166–73. doi: 10.1016/j.ajem.2021.12.012, PMID: 34923196 PMC8665665

[8] Karasneh RA Khassawneh BY Al-Azzam S Al-Mistarehi A-H Lattyak WJ Aldiab M. Risk factors associated with mortality in COVID-19 hospitalized patients: data from the Middle East. Int J Clin Pract. (2022) 2022:1–10. doi: 10.1155/2022/9617319, PMID: 36072822 PMC9398873

[9] Al-Mistarehi AH Al-Azzam S Karasneh R Al Sbihi A Alomari S Haj Hussein AA. Risk factors for in-hospital mortality among adults hospitalized with COVID-19: a cross-sectional study from Jordan. Chest. (2022) 162:A659–60. doi: 10.1016/j.chest.2022.08.514

[10] Kabbaha S Al-Azzam S Karasneh RA Khassawneh BY Al-Mistarehi AH Lattyak WJ. Predictors of invasive mechanical ventilation in hospitalized COVID-19 patients: a retrospective study from Jordan. Expert Rev Respir Med. (2022) 16:945–52. doi: 10.1080/17476348.2022.2108796, PMID: 35929952

[11] Cheng S Zhao Y Wang F Chen Y Kaminga AC Xu H. Comorbidities' potential impacts on severe and non-severe patients with COVID-19: a systematic review and meta-analysis. Medicine (Baltimore). (2021) 100:e24971. doi: 10.1097/MD.0000000000024971, PMID: 33761654 PMC9281964

[12] Mohan AA Olson LB Naqvi IA Morrison SA Kraft BD Chen L. Age and comorbidities predict COVID-19 outcome, regardless of innate immune response severity: a single institutional cohort study. Crit Care Explor. (2022) 4:e0799. doi: 10.1097/CCE.0000000000000799, PMID: 36506827 PMC9726311

[13] Toofan F Hosseini SM Alimohammadzadeh K Jafari M Bahadori M. Impact of comorbidities on mortality in hospitalized patients with COVID-19: an experience from Iran. J Educ Health Promot. (2021) 10:460. doi: 10.4103/jehp.jehp_1589_20, PMID: 35233407 PMC8826893

[14] Singh P Bhaskar Y Verma P Rana S Goel P Kumar S. Impact of comorbidity on patients with COVID-19 in India: a nationwide analysis. Front Public Health. (2023) 10:1027312. doi: 10.3389/fpubh.2022.1027312, PMID: 36777781 PMC9911546

[15] Roedl K Jarczak D Boenisch O de Heer G Burdelski C Frings D. Chronic critical illness in patients with COVID-19: characteristics and outcome of prolonged intensive care therapy. J Clin Med. (2022) 11:1049. doi: 10.3390/jcm11041049, PMID: 35207322 PMC8876562

[16] Carvalho PR Sirois P Fernandes PD. The role of kallikrein-kinin and renin-angiotensin systems in COVID-19 infection. Peptides. (2021) 135:170428. doi: 10.1016/j.peptides.2020.170428, PMID: 33065209 PMC7553876

[17] Ángeles Correa MG Villarreal Ríos E Galicia Rodríguez L Vargas Daza ER Frontana Vázquez G Monrroy Amaro SJ. Enfermedades crónicas degenerativas como factor de riesgo de letalidad por COVID-19 en México [Chronic degenerative conditions as risk factors for lethal COVID-19Doenças crônicas degenerativas como fator de risco para letalidade por COVID-19]. Rev Panam Salud Publica. (2022) 46:e40. doi: 10.26633/RPSP.2022.40, PMID: 35509641 PMC9060181

[18] Peña JE Rascón-Pacheco RA Ascencio-Montiel IJ González-Figueroa E Fernández-Gárate JE Medina-Gómez OS. Hypertension, diabetes and obesity, major risk factors for death in patients with COVID-19 in Mexico. Arch Med Res. (2021) 52:443–9. doi: 10.1016/j.arcmed.2020.12.002, PMID: 33380361 PMC7832055

[19] Siqueira LD Muchon JD Arruda JT Paludo RL d R. Analysis of mortality from COVID-19 and obesity as a risk factor. RSD. (2022) 11:e10911123432. doi: 10.33448/rsd-v11i1.23432

[20] Das SKRA Birangal SR Nikam AN Pandey A Mutalik S Joseph A. Role of comorbidities like diabetes on severe acute respiratory syndrome coronavirus-2: a review. Life Sci. (2020) 258:118202. doi: 10.1016/j.lfs.2020.118202, PMID: 32758625 PMC7397991

[21] Sindi AA Tashkandi WA Jastaniah MW Bashanfar MA Fakhri AF Alsallum FS. Impact of diabetes mellitus and co-morbidities on mortality in patients with COVID-19: a single-center retrospective study. Saudi Med J. (2023) 44:67–73. doi: 10.15537/smj.2023.44.1.20220462, PMID: 36634951 PMC9987682

[22] Nagy É Cseh V Barcs I Ludwig E. The impact of comorbidities and obesity on the severity and outcome of COVID-19 in hospitalized patients-a retrospective study in a Hungarian hospital. Int J Environ Res Public Health. (2023) 20:1372. doi: 10.3390/ijerph20021372, PMID: 36674133 PMC9859007

[23] Pranata R Henrina J Raffaello WM Lawrensia S Huang I. Diabetes and COVID-19: the past, the present, and the future. Metabolism. (2021) 121:154814. doi: 10.1016/j.metabol.2021.154814, PMID: 34119537 PMC8192264

[24] Rocha GV Soares CEM Filho LH d O do AMVF de Castro VE Junior EA. A influência da obesidade na mortalidade de adultos com COVID-19 / the influence of obesity on adult mortality with COVID-19. Braz J Hea Rev. (2021) 4:1405–18. doi: 10.34119/bjhrv4n1-119

[25] Suresh S Siddiqui M Abu Ghanimeh M Jou J Simmer S Mendiratta V. Association of obesity with illness severity in hospitalized patients with COVID-19: a retrospective cohort study. Obes Res Clin Pract. (2021) 15:172–6. doi: 10.1016/j.orcp.2021.02.006, PMID: 33653666 PMC7904471

[26] Ribeiro AC Poli P da SA USC. Increased risk of mortality from COVID-19 in people with obesity. Rev Rene. (2023) 24:e81453. doi: 10.15253/2175-6783.20232481453

[27] Belchior-Bezerra M Lima RS Medeiros NI Gomes JAS. COVID-19, obesity, and immune response 2 years after the pandemic: a timeline of scientific advances. Obes Rev. (2022) 23:e13496. doi: 10.1111/obr.13496, PMID: 35837843 PMC9349458

[28] Bornstein SR Rubino F Khunti K Mingrone G Hopkins D Birkenfeld AL. Practical recommendations for the management of diabetes in patients with COVID-19. Lancet Diabetes Endocrinol. (2020) 8:546–50. doi: 10.1016/S2213-8587(20)30152-2, PMID: 32334646 PMC7180013

